# The associations between malaria, interventions, and the environment: a systematic review and meta-analysis

**DOI:** 10.1186/s12936-018-2220-x

**Published:** 2018-02-07

**Authors:** Margaux L. Sadoine, Audrey Smargiassi, Valéry Ridde, Lucy S. Tusting, Kate Zinszer

**Affiliations:** 1 Université de Montréal Public Health Research Institute (Institut de Recherche en Santé Publique (IRSPUM)), Université de Montréal, Montréal, QC Canada; 2 School of Public Health, Department of Social and Preventive Medicine, Université de Montréal, Montréal, QC Canada; 3 School of Public Health, Department of Environmental and Occupational Health, Université de Montréal, Montréal, QC Canada; 4 Institut national de santé publique du Québec, Montréal, QC Canada; 5 Oxford Big Data Institute, Li Ka Shing Centre for Health Information and Discovery, University of Oxford, Oxford, UK

**Keywords:** Malaria, Climate, Environment, Systematic review, Meta-analysis, Prediction, Malaria control, Interventions, Bed nets

## Abstract

**Background:**

Malaria transmission is driven by multiple factors, including complex and multifaceted connections between malaria transmission, socioeconomic conditions, climate and interventions. Forecasting models should account for all significant drivers of malaria incidence although it is first necessary to understand the relationship between malaria burden and the various determinants of risk to inform the development of forecasting models. In this study, the associations between malaria risk, environmental factors, and interventions were evaluated through a systematic review.

**Methods:**

Five electronic databases (CAB Abstracts, EMBASE, Global Health, MEDLINE and ProQuest Dissertations & Theses) were searched for studies that included both the effects of the environment and interventions on malaria within the same statistical model. Studies were restricted to quantitative analyses and health outcomes of malaria mortality or morbidity, outbreaks, or transmission suitability. Meta-analyses were conducted on a subset of results using random-effects models.

**Results:**

Eleven studies of 2248 potentially relevant articles that met inclusion criteria were identified for the systematic review and two meta-analyses based upon five results each were performed. Normalized Difference Vegetation Index was not found to be statistically significant associated with malaria with a pooled OR of 1.10 (95% CI 0.07, 1.71). Bed net ownership was statistically associated with decreasing risk of malaria, when controlling for the effects of environment with a pooled OR of 0.75 (95% CI 0.60, 0.95). In general, environmental effects on malaria, while controlling for the effect of interventions, were variable and showed no particular pattern. Bed nets ownership, use and distribution, have a significant protective effect while controlling for environmental variables.

**Conclusions:**

There are a limited number of studies which have simultaneously evaluated both environmental and interventional effects on malaria risk. Poor statistical reporting and a lack of common metrics were important challenges for this review, which must be addressed to ensure reproducibility and quality research. A comprehensive or inclusive approach to identifying malaria determinants using standardized indicators would allow for a better understanding of its epidemiology, which is crucial to improve future malaria risk estimations.

**Electronic supplementary material:**

The online version of this article (10.1186/s12936-018-2220-x) contains supplementary material, which is available to authorized users.

## Background

Vector-borne diseases are particularly vulnerable to climate change with malaria being the most prevalent. Malaria is currently endemic in 91 countries representing 3.2 billion people at-risk, nearly half of the world’s population [1]. In 2015, the World Health Organization estimated 214 million cases of malaria and 438,000 deaths, with more than two-thirds (70%) of all malaria deaths occurring in children under five [1]. Malaria is endemic in tropical and subtropical climatic regions of the world including Africa, Asia, Central and South America and certain Caribbean islands [2]. These regions are highly conducive to malaria transmission given the temperature and humidity needs of the *Anopheles* mosquitoes and *Plasmodium* parasites [2].

The relationship between climatic or meteorological conditions, such as temperature and rainfall, and the mosquito have been well documented [3–13]. Despite this, there is debate in the scientific literature surrounding the implications of climate change on malaria transmission and future disease burden [14–20]. The controversy arises given the complex and multifaceted connections between malaria transmission, socioeconomic conditions, climate, and other environmental factors. Socio-economic development including improved living conditions, vector control interventions, and effective treatment measures are cited as protective effects against malaria and which likely moderate the relationship between climate and malaria [9, 21, 22]. Conversely, increased insecticide resistance, land use changes, population mobility and population growth with inadequate housing are associated with rising incidence and also likely influence the relationship between climate and malaria [2, 16, 23, 24].

There has been tremendous advancement in malaria control and prevention, with an estimated 663 million clinical cases averted between 2000 and 2015 in Africa [25]. The main contributors to this reduction are attributed to insecticide-treated bed nets (68%), artemisinin-based combination therapy (ACT, 22%), and insecticide residual spraying (IRS, 10%) [25]. Currently, bed nets are the primary prevention strategies [26]. Insecticide-treated nets (ITNs) have been demonstrated to reduce the occurrence of malaria episodes, all-cause child mortality, and complications associated with malaria during pregnancy [26, 27]. Bed nets are often accessible through mass distribution campaign and health facilities where pregnant women and children under age five are the priority targets, and receive the nets free of cost during routine antenatal care and routine immunization visits [26–31]. Reductions in malaria incidence from ACT use are due to the prevention of severe disease and death [25] while IRS is highly effective at rapidly eliminating adult *Anopheles* mosquitoes [32]. The effectiveness of IRS largely depends on adequate program capacity and requires high household coverage (e.g., more than 85%) [32].

The magnitude of resources committed to malaria control is enormous and accurate forecasting modelling would greatly assist clinical and public health services in providing lead time to organize targeted, proactive responses. Prediction of the future malaria burden is typically based upon few parameters, such as temperature and rainfall, which is limited and forecasting models should take into account non-climatic factors, such as socio-economic development and intervention measures [14, 17, 19, 21, 33, 34]. Before forecasting models can be developed, it is imperative to understand the relationship between malaria burden and the various determinants of risk to identify important drivers. In this study, associations between malaria risk, environmental factors, and interventions have been systematically evaluated.

## Methods

A systematic review was conducted to characterize the literature and a meta-analysis was performed to assess the strength of associations and quality of the data. Recommendations of the preferred reporting items for systematic reviews and meta-analysis were followed [35]. The study is registered with the International Prospective Register of Systematic Reviews (CRD 42017062593) [36].

### Eligibility criteria

The searches were not restricted by year or country but by language, as only English and French literature was selected. Only original research studies with quantitative analysis were considered, thereby excluding reviews, short communications, letters, posters, and conference abstracts. Studies were included if the analysis took into account both the effects of environmental factors and malaria control interventions within the same statistical model. Environment was considered as a generic term covering both climatic and meteorological concepts and includes meteorological data and remote sensing data captured from satellites. The intervention term encompassed a wide range of measures related to malaria control including vector control (e.g., bed nets, spraying, larval control), medical treatment for malaria, health services (accessibility and research), health practices and knowledge, community health workers, health education and promotion, health policy, and surveillance. Studies were included if the health outcome was based upon malaria mortality or morbidity, malaria outbreaks, or malaria transmission suitability. Studies were excluded if they did not consider environmental factors and interventions within the same model, as it was expected that the effects of interventions on malaria risk would differ depending on the meteorological (e.g., temperature or precipitation) conditions, and that the effects of meteorological conditions on malaria risk would also vary with intervention effects. Finally, studies were excluded if the models were based upon mosquito vector populations and if the model was a simulation or mathematical model (not based upon empirical data).

### Search strategy

Papers were identified using medical subject headings and key word combinations and truncations, and the following categories were combined using the AND Boolean logic operator: (i) malaria terms, malaria intervention terms, and environmental terms (see Additional file 1). The citation searches began on 11 February 2016 and the final citation search was conducted on 28 March 2017. We searched the following databases: CAB Abstracts (1910–2016 week 4), EMBASE (1947–2016 10 February), Global Health (1973–2016 week 4), MEDLINE (1946–2016 11 February) and ProQuest Dissertations & Theses databases (1982–15 February 2016). The citations were imported into EndNote X7.4 (Thomas Reuters) for management. Two main reviewers (MS and KZ) examined all citations in the study selection process. The first stage of review involved each reviewer independently identifying potentially relevant studies based upon information provided in the title and abstract. If it was uncertain whether to include a study, the citation was kept and included for full article review. The second stage of review involved each reviewer independently identifying relevant studies based upon full article review. A third reviewer (AS) was consulted when there was discordance between the two reviewers.

### Data extraction

From each selected study, the following information were abstracted: references, country, population setting, outcome, environmental data, time-frame of observed data, interventions, analytical approach, results and limitations. Extracted data were entered into an Excel table. To evaluate how well the interventions were described in the studies, the 12-item checklist of the TIDieR (template for intervention description and replication) [37] was applied: name of intervention, why, what (materials), what (procedure), who provided, how, where, when and who much, tailoring, modifications, how well (planned), and how well (actual). All study authors were contacted for further information.

### Quality of evidence

A quality assessment guide was developed, adapted from the NHLBI (National Heart, Lung, and Blood Institute) quality assessment tool [38], to fit the study designs of the selected publications. The questions were developed to evaluate internal and external validity which is also available in Additional file 2.

### Meta-analysis

A quantitative pooling of results was undertaken to perform a meta-analysis with Review Manager 5.3. Given the heterogeneity of measures for the same concept or indicator across the studies, the analysis was performed for the most common intervention and environmental concept. The intervention and environmental meta-analysis were performed separately using the generic inverse variance method, which assigns each effect a weight equal to the inverse of its variance [23]. Pooled ORs were calculated using random effects in the meta-analyses.

## Results

**Fig. 1:**
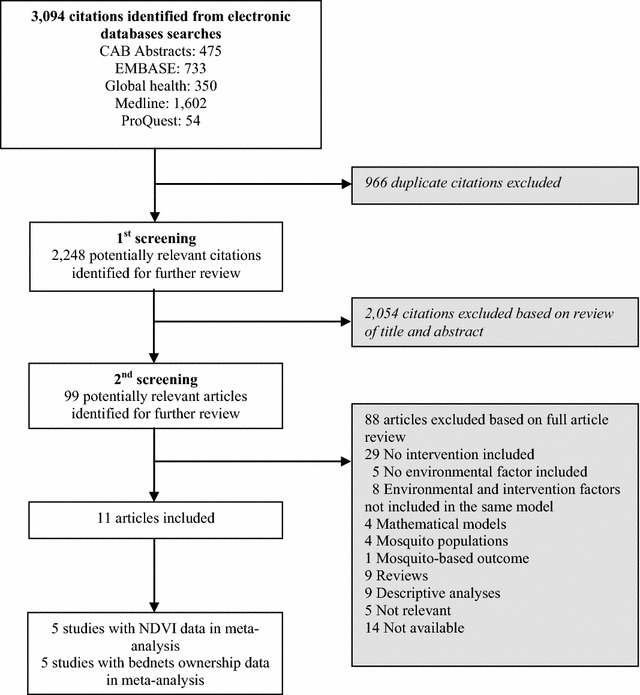
Flow of literature search

From the search, 2248 potentially relevant articles were identified for the systematic review after duplicate citations were removed (Fig. 1). After abstract review, 99 articles were selected and from these, 42 were removed as they either did not consider the effect of intervention or environmental factors or they did not consider these determinants in same models. Eleven studies out of 2248 that met inclusion criteria were identified for this review (Additional file 3). The majority of research was conducted in African countries (10 of 11 studies) with one publication based in South Asia (Nepal). Seven of the articles provided effect estimates at the national level [39–45], while two studies were at the district level [46, 47], one at village level [48], and one at both national and district levels. All articles were published between 1999 and 2016.

### Malaria indicators

Malaria prevalence, parasitaemia, and incidence were the three different indicators of malaria risk (Additional file 3). The majority of the studies used laboratory-confirmed case data [39–43, 45, 46, 48, 49] although two studies used clinically diagnosed cases [44, 47].

### Intervention indicators

The most common intervention included was bed nets (eleven studies [39–49]), followed by IRS (six studies [40, 43, 45–47, 49]), larval control (one study [47]), ACT (one study [49]). Six studies included more than one intervention [40, 43, 45–47, 49]. A variety of metrics were used as bed net indicators at the at the household or community level: access to ITN in household [39], ITN/net use [41, 48, 49], ownership of ITN/net [40, 43], presence of at least one bed net in the household [45] or per two household members [42], ITN coverage/distribution rate [44, 46], and the numbers of new impregnated nets distributed and old nets reimpregnated [47]. The majority of studies (n = 7) used data from the standardized household Malaria Indicator Survey [39–43, 45, 49]. The remaining studies obtained intervention data from their Ministry of Health or the National Malarial Control Programme [44, 46–48].

### Intervention content

**Fig. 2:**
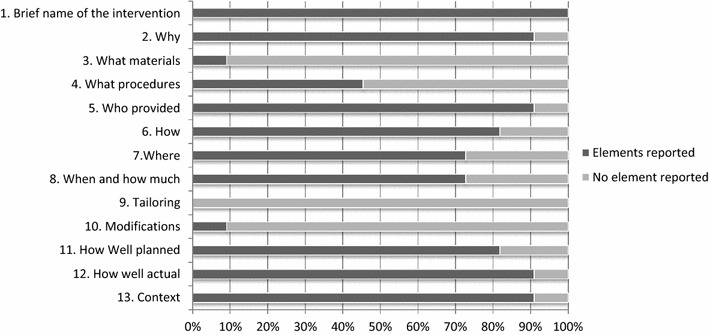
Percentage of studies with elements reported per TIDieR item

Based upon the TIDieR checklist, comprehensive intervention reporting was extremely poor for ten of the eleven studies. Details on implementation, procedure, the mobilized staff and agents, monitoring of interventions were not provided or were but with minimal information. Only one study [46] provided details regarding the period of implementation, amount of insecticide sprayed, and information regarding policy changes about intervention coverage and suppliers. TIDieR tables (Additional file 4) were completed based on external searches as publications information provided in the articles was insufficient. The percentages of studies with data according to TIDieR items are presented in Fig. 2. Details of interventions are available on Additional file 3.

### Environmental data

Climatic variables analyzed were rainfall and humidity, while environmental variables were represented by vegetation index (enhanced or normalized, which is a vegetation greening indicator) and temperature (air and land surface). The majority of studies obtained environmental data from remote sensing satellites [39, 40, 42–45, 47–49]. The measures for the different indicators were very heterogeneous across the studies. For example, there were six different measurements of rainfall: annual average, 3 month average, monthly average, 20 day cumulative, and daily estimates. Temperature and vegetation index were also variably measured (Additional file 3).

### Study design and analytical approach

Selected studies can be classified into three categories: ecological, where the variables are measured at the population or area level rather than individual-level [44, 46, 47], cross-sectional [48] and quasi cross-sectional [39–43, 45, 49] studies. Quasi cross-sectional studies correspond to cross-sectional studies where the outcome and other covariates are measured at the individual-level while the exposure of interest (e.g., climate) is measured at the population-level. The associations between malaria risk, the environment, and interventions were evaluated using geostatistical or generalized linear models adjusted for spatial correlation in nine studies [39–44, 46, 48, 49], and two studies having used Poisson and multivariate logistic regressions [45, 47].

### Quality

Overall, the studies were of medium or good quality. Specifically, six studies were evaluated to be of medium quality [41, 44–48], and five of good quality [39, 40, 42, 43, 49]. Medium quality studies are characterized by a lower reliability of the data (i.e., based upon surveillance data), and/or lower validity of the outcome measure (i.e., clinical confirmation of malaria cases) and/or lack of the inclusion of important confounding variables, and/or poor or unclear reporting of statistical findings. All good quality studies accounted for major confounding factors and generally used valid and reliable data (e.g., Malaria Indicator Survey). Rating details are provided in Additional file 2.

### Association between the environment, interventions, and malaria

The results from the studies are summarized in Tables 1 and 2. A significant protective effect of bed nets (ownership, use, distribution) was largely found while controlling for environmental variables [40–42, 45–49] (Table 1). IRS was mainly found to be not statistically associated with malaria risk [43, 45, 46, 49].

**Table 1:** Summary of the point estimates characterizing the association of malaria risk with malaria control interventions

	Country	Effect measure	Indicator	ITN or nets (95% CI)	IRS (95% CI)	ACT (95% CI)
Adigun [39]	Nigeria	OR	Proportion with access to ITN in the household	0.86 (0.51, 1.48)		
Bennett [40]	Zambia	OR	ITN ownership	0.74 (0.64, 0.86)	0.30 (0.18, 0.51)	
Chirombo [41]	Malawi	OR	ITN use	0.57 (0.43, 0.76)		
Dhimal [46]	Nepal	RR	LLIN coverage	0.75 (0.62, 0.92)	NR	
Diboulo [49]^a^	Burkina Fasso	OR	Bednet use	1.66 (0.89, 3.08)	1.14 (0.17, 7.23)	1.45 (0.49, 4.21)
Diboulo [49]^a^	Burkina Fasso	OR	Bednet use	0.25 (− 0.37, 0.90)	0.11 (− 1.75, 1.70)	0.13 (− 1.49, 1.67)
Giardina [42]	Senegal	OR	Presence of at least one bed net per 2 HH members	0.14 (0.03, 0.7)		
Gosoniu [43]	Tanzania	OR	Bednet ownership	0.92 (0.75, 1.13)	1.17 (0.33, 3.63)	
Graves [47]^b^	Eritrea	NS	Monthly numbers of new and old nets impregnated	1.00	1.00 (per kg of DDT) 1.00 (per kg of Malathion)	
Graves [47]^b^	Eritrea	NS	Monthly numbers of new and old nets impregnated	1.00	NR	
Lowe [44]	Malawi	OR	ITN distribution rate	NR		
Riedel [45]	Zambia	OR	Presence of at least one bed net in HH	0.60 (0.39, 0.88)	1.73 (0.42, 6.90)	
Thomson [48]	Gambia	NS	Bednet use	0.51		

*NR* not reported, *NS* not specified, *OR* odd ratio, *RR* risk ratio

^a^Diboulo et al. studied interventions at national and district level

^b^Graves et al. studied interventions in two different areas (Gash Barka Zoba and Anseba Zoba)

**Table 2:** Summary of point estimates characterizing the association of malaria risk with environmental factors

	Country	Effect measure	Rainfall indicator	Rainfall (95% CI)	Vegetation indicator	Vegetation index	Temperature indicator	Temperature	Humidity
Adigun [39]	Nigeria	OR	Decadal rainfall (mm)	0.72 (0.57, 0. 91)	NDVI	1.56 (1.21, 1.99)			
Bennett [40]	Zambia	OR	20 days rainfall (mm)	2.04 (1.38, 3.00)	EVI	1.98 (1.48, 2.65)			
Chirombo [41]	Malawi	OR	Mean rainfall (mm/day)	NR			3 months min temp (°C)	NR	
Dhimal [46]	Nepal	RR					Monthly min temp (°C)	1.27 (1.12, 1.45)	0.91 (0.83, 1.00)
Diboulo [49]^a^ (National level)	Burkina Fasso	OR					8 days NLST (°C)	0.81 (0.72, 0.90)	
Diboulo [49]^a^ (District level)	Burkina Fasso	OR					8 days NLST (°C)	0.82 (0.72, 0.93)	
Giardina [42]	Senegal	OR			NDVI	0.91 (0.61, 1.83)	Weekly NLST (°C)	0.83 (0.53, 1.26)	
Gosoniu [43]^b^	Tanzania	OR	Annual average (15–20 mm)	0.97 (0.48, 1.90)	NDVI (0.4–0.6)	1.47 (0.88, 2.45)	Annual average night temp (16–20 °C)	1.47 (0.81, 2.73)	
Gosoniu [43]^b^	Tazania	OR	Annual average (20 mm)	1.43 (0.63, 3.14)	NDVI (0.6)	1.40 (0.67, 2.98)	Annual average night temp (20 °C)	1.31 (0.61, 2.81)	
Graves [47]^c^ (Gash Barka *Zoba*)	Eritrea	NS	Monthly precipitation (mm/day)	1.00	NDVI	4.73			
Graves [47]^c^ (Anseba *Zoba*)	Eritrea	NS	Monthly precipitation (mm/day)	1.00	NDVI	14 × 10^3^			
Lowe [44]	Malawi	OR	Monthly precipitation (mm/day)	NR			Temp estimates (°C)	NR	
Riedel [45]	Zambia	OR	Daily rainfall estimate (mm)	1.21 (0.85, 1.68)	NDVI	1.28 (0.67, 2.73)	8 days NLST (K)	1.21 (0.77, 1.88)	
Thomson [48]	Gambia	NS			NDVI	0.67			

*NR* not reported, *NS* not specified, *OR* odd ratio, *RR* risk ratio

^a^Diboulo et al. studied environmental drivers at national and district level

^b^Gosoniu et al. studied different measures of rainfall, vegetation index and temperature indicators

^c^Graves et al. studied environmental drivers in two different areas (Gash Barka Zoba and Anseba Zoba)

In terms of environmental effects, findings were not consistent; four studies did not detect a significant association with malaria, while seven showed positive or negative associations with environmental variables (Table 2). While controlling for the effect of interventions, rainfall was almost equally associated with an increasing risk of malaria [40, 44, 47] as not significantly associated with malaria risk [41, 43, 45]. Likewise, vegetation index was found to be closely associated with malaria [39, 40, 47] as well as not associated [42, 43, 45]. However, temperature was mainly not statistically associated with malaria risk [41–45]. Relative humidity was analyzed in only one study and was statistically associated with an increasing risk of malaria [46].

### Meta-analysis

**Fig. 3:**
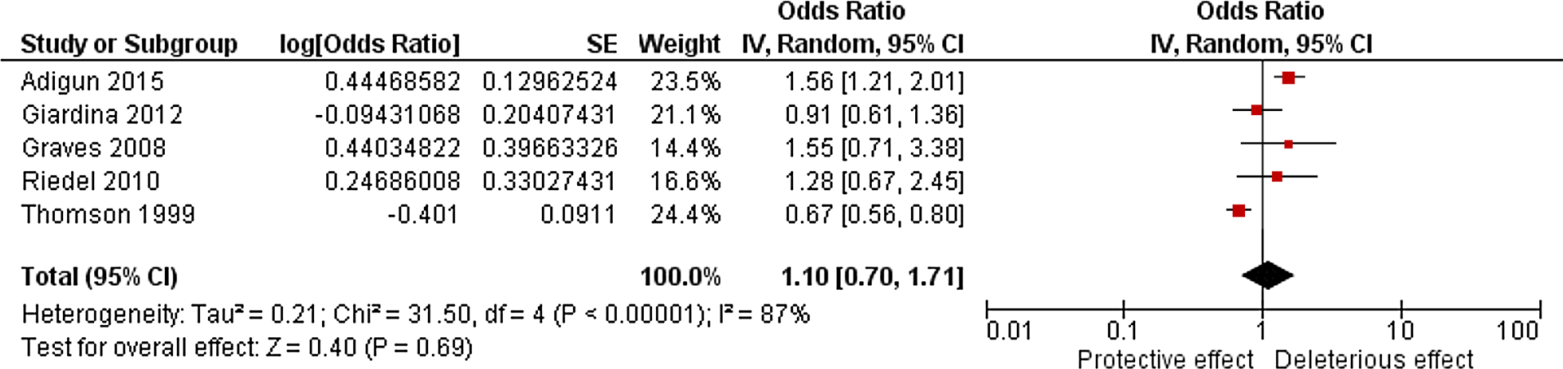
Meta-analysis of the association between malaria risk and NDVI. Pooled effects from random-effects meta-analyses for adjusted results are shown

Given the small number of studies and the disparate measures across the studies, the most common indicators were considered for the meta-analysis. Rainfall and temperature indicators could not be pooled given the measurement heterogeneity. There was large variation observed for bed nets indicators including household use [41, 48, 49], household ownership [39, 40, 42, 43, 45], distribution [44, 46], or household number of impregnated nets [47]. Given these restrictions, only the effects of bed net ownership and NDVI were analyzed. NDVI was not statistically associated with malaria while controlling for the effect of intervention (OR 1.10, 95% CI 0.70, 1.71), although the heterogeneity across the studies was very high (I^2^ = 87%), indicating an unstable and unreliable pooled estimate. Five studies [39, 42, 45, 47, 48] were included in the NDVI analysis, among which three studies did not report significant associations between malaria risk and NDVI (Fig. 3).

**Fig. 4:**
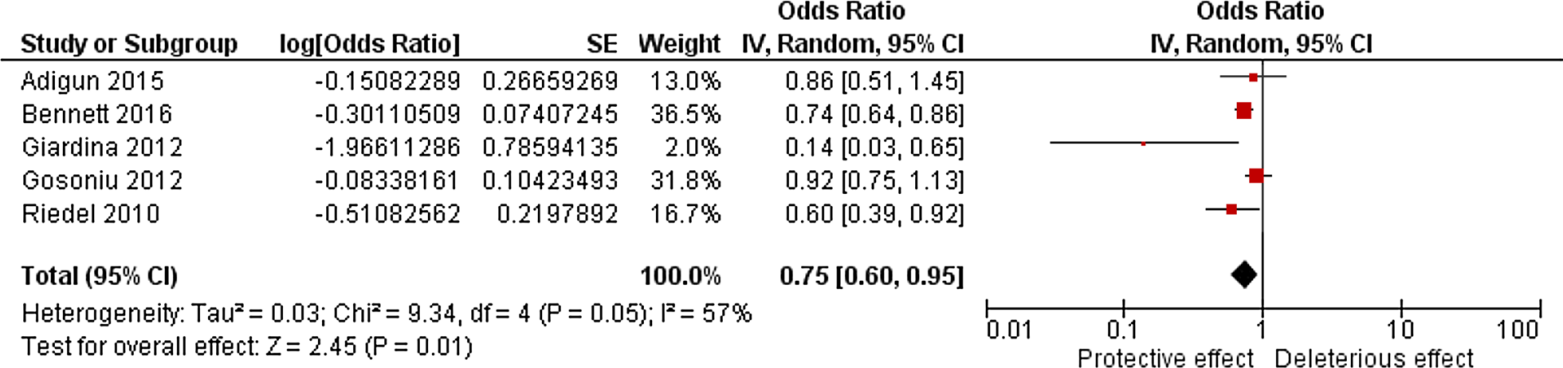
Meta-analysis of the association between bed net ownership and malaria risk. Pooled effects from random-effects meta-analyses for adjusted results are shown

The analysis of bed net ownership, while controlling for the effect of environment, was also performed with five studies [39, 40, 42, 43] and demonstrated a significant protective effect (OR 0.75, 95% CI 0.60, 0.95) with a relative high heterogeneity (I^2^ = 57%). Two of the five included studies presented no significant association (Fig. 4).

## Discussion

The heterogeneity of how environmental and intervention variables are measured, creates important challenges for pooling data across studies and also to infer study findings. In this review, the environmental influence on malaria risk was inconsistent for rainfall, temperature, and NDVI between the studies although generally, they were more often associated with increased risk. Bed net ownership was the most common intervention included in studies and found to have a protective effect on malaria in almost every included study, while other interventions such as IRS and ACT were more varied, although not often included. Additionally, there are a very limited number of studies that have examined the environmental and interventional effects on malaria risk, despite the importance and influence of both.

The meta-analysis showed no effect of NDVI, although this is associated with a very high heterogeneity measure due to the low number of studies included in the meta-analysis. The difference in outcome measurement and included confounders, the presence of residual confounding, and different analytic approaches may explain the inconsistency in the association between NDVI and malaria risk between the studies. The pooled estimate of bed net ownership showed that bed nets ownership is statistically associated with a modest decrease in malaria risk. However, it must be noted that ownership indicators do not capture actual bed net use [49]. In this review, studies were selected to control for both the effect of interventions and the environment on malaria risk. However, very few studies simultaneously analysed both types of variables; only 0.5% of all potentially relevant studies (11/2248) included both interventions and environmental determinants within the same statistical model—none evaluated an interaction effect between the environment and intervention. Examining the interaction between environmental factors and intervention on malaria risk would provide valuable information, for example, in how the association between malaria risk and bed net changes for different levels of rainfall. Furthermore, there was large variation in the measures across all studies including seven different measures of temperature, six for rainfall, seven different indicators for bed nets, which can partly explain the variability of findings.

Studies included in this review were often of medium quality (n = 6) and there was a poor reporting of statistics and intervention information. Point estimates were not systematically provided, neither were confidence intervals and standard errors. There is a need to standardize the reporting of results across journals to ensure reproducibility and quality research. Only a few studies provided complete descriptions of the environmental data; often it was not possible to determine if the measures were averages or represented a unique measure during a specific point in time. Also, almost none of the study provided enough material to understand the context of the interventions, timing, how, and where they were deployed, which is crucial to understanding the processes of implementation and the effectiveness of interventions [50, 51]. This raises concerns for reproducibility once again but also for portability and repeatability of interventions for field actors. There is a need to “contextualize” intervention research to identify and understand underlying conditions that contribute to systematic differences in population health status [51]. The TIDieR checklist could be an easy and useful tool to help researchers strengthen the quality of studies and to provide a systematic approach for intervention description.

This review was limited by the low number of studies included in addition to the high heterogeneity of measures between these studies. The research was restricted to French and English while a certain number of publications were published in Spanish, Portuguese and Chinese on this topic, which were not considered for this review. In addition, grey literature was not reviewed for any relevant studies and the selection was restricted on studies that analysed both the effects of environmental factors and malaria control interventions in the same model.

There are systematic reviews including meta-analyses on insecticide treated nets and indoor residual spraying, which indicate protective effects of insecticide treated bed nets and to a lesser extent, indoor residual spraying [52–54]. This body of work supports the notion of including these interventions when predicting malaria, given their significant associations with malaria risk. Zhang and Hiller reviewed the relationship between climate variability and the transmission of vector-borne diseases, including malaria, and found that the quantitative relationship between climate and vector-borne diseases was inconsistent across studies [55]. Many articles were found to be methodologically limited, for example, not properly adjusting for the autocorrelation of variables in the models, also because of availability and quality of health and climatic variables. Reiner et al., systematically reviewed the seasonality of *Plasmodium falciparum* and reported important differences between studies, relative to definition of metrics and a lack of consistency in the approach used across studies (i.e. different spatial and temporal scales), making difficult to summarize the findings [3]. Malaria transmission varies widely within and across countries as the micro-epidemiological variation of malaria is related to fine-scale heterogeneity in environmental, genetic, social, and other contextual factors [56].

In this view, recommendations include that more comprehensive research is needed, when examining the determinants of malaria and should include interventions, environmental factors, and socio-demographics, which would allow a better understanding of malaria epidemiology and also identify important predictors to consider for forecasting work. Various environmental and population settings should be explored to improve the understanding of contextual contributions to malaria risk. Standardization of indicators would ensure improved comparability between studies as well as a common approach to reporting the results, which should minimally consist of effect measures and associated confidence intervals for all variables included in a model. Full description of model development and evaluation should be stated as well. Environmental variables that have been shown to be the most related or the most consistently related to malaria risk should be systematically used in future studies assessing the effects of climate (and of interventions). This will allow for the pooling of data and future work should also include clear descriptions of variables and categorizations, considerations of timescale, units of measurement, and lagged effects. A comprehensive and systematic description of interventions is strongly suggested to better understand the types of interventions studied, and importantly, to enable appropriate comparisons and conclusions. Having a detailed description of interventions, context, and actors involved are necessary to explain the failure or effectiveness of an intervention [50, 51].

## Additional files

**Additional file 1.** Ovid Medline search.

**Additional file 2.** Quality Assessment guide.

**Additional file 3.** Characteristics of studies included in the review (n = 11).

**Additional file 4.** TIDieR checklist.

## Abbreviations

ACT: artemisinin-based combination therapy
CI: confidence intervals
IRS: indoor residual spraying
ITN: insecticide-treated net
NDVI: Normalized Difference Vegetation Index
NLST: night land surface temperature
NR: not reported
NS: not specified
OR: odds ratio
RDT: rapid diagnostic test
RR: risk ratio
