# Supplementary material for: The associations between malaria, interventions, and the environment: a systematic review and meta-analysis
Source: Malar J. 2018 Feb 7;17:73. doi: 10.1186/s12936-018-2220-x (PMC5803989; doi:10.1186/s12936-018-2220-x)
Supplement: Supplementary file 1 — Additional file 1. Ovid Medline search. [file 12936_2018_2220_MOESM1_ESM.docx]

**Additional File 1**

**Ovid Medline search**

1. exp Malaria/
2. (malaria* or paludism or plasmodium or marsh fever).tw.
3. exp Climate/ or exp Climate Change/
4. (climate or climate change or climate variables or (“climate” and “change”)).tw.
5. (malaria control or malaria intervention* or malaria control measures or vector control* or vector management).tw.
6. exp Insecticide-treated bednets/
7. (bednet* or net* or ITN* or insecticide-treat* net or insecticidal-treat* net or insecticide-net or insecticidal-net or bed-net or bednet or treated-net or mosquito-net or LLIN* or long-last* net). tw.
8. (IRS or indoor-residual spray* or indoor-spray* or spray*).tw.
9. (Source reduction).tw.
10. (larvicid* or larva* control).tw.
11. exp Antimalarials/
12. (antimalaria* or anti-malaria* or intermittent preventive treatment* or intermittent preventive therap* or ITP* or artemisinin-based combination therap* ).tw.
13. (diagnostics or microscop* or rapid diagnos* test* or rapid test* or RDT*).tw.
14. exp “fees and charges”/
15. exp Health services accessibility/
16. exp Health policy/
17. exp Community health workers/
18. exp health services research/
19. (community health worker* or health service* access* or access* or train* or health service* research* or health polic*).tw.
20. exp Case management/
21. exp Forecast*/ and exp Models, Theoretical/
22. exp Public Health Surveillance/
23. exp surveillance/
24. exp health information system/
25. (surveillance or early warning system* or health information system).tw.
26. exp health promotion/
27. exp health education/
28. exp Health knowledge, attitude, practice/
29. (health promotion or health education or health knowledge or health attitude or health practice).tw.
