# Supplementary material for: The associations between malaria, interventions, and the environment: a systematic review and meta-analysis
Source: Malar J. 2018 Feb 7;17:73. doi: 10.1186/s12936-018-2220-x (PMC5803989; doi:10.1186/s12936-018-2220-x)
Supplement: Supplementary file 2 — Additional file 2. Quality Assessment guide. [file 12936_2018_2220_MOESM2_ESM.docx]

**Additional File 2 - Quality Assessment guide**

| **Criteria** | **Notation** | | | **Maximum points attributable** |
| --- | --- | --- | --- | --- |
| 1. Was the research question or objective in this paper clearly stated? | Yes=1  No=0 | | | 1 |
| 2. Were the data collection methods clearly specified (MIS* vs. surveillance)? | MIS=2 Surveillance=1 | | | 2 |
| 3. Was variance and adjusted effect estimates provided? | **Adjusted effect estimate**  Env** and intervention = 1  Env** or intervention = 0.5  No=0 | | **Variance**  Yes = 1 No = 0 | 2 |
| 4. Were the outcome measures clearly defined (e.g., malaria parasitema), valid (e.g., lab confirmed using microscopy), and reliable (e.g., obtained from surveillance or MIS*)? | **Defined**  Yes = 1 No/unclear = 0 | **Valid**  Lab confirmed = 1 Not lab confirmed = 0 | **Reliable**  MIS* = 1  Surveillance = 0.5 | 3 |
| 5. Were the intervention measures clearly defined (e.g., bednet possession in household), valid (e.g., self-report vs visual inspection) and reliable (e.g., obtained from surveillance or MIS*)? | **Defined**  Yes = 1 No/unclear = 0 | **Valid**  Visual inspection = 1  Self-reported = 0.5  Unclear = 0 | **Reliable**  MIS*=1  Surveillance = 0.5 | 3 |
| 6. Was the exposure measures clearly defined (e.g., cumulative weekly rainfall in mm), valid (e.g., met station vs. remote sensing) and reliable? | **Defined**  Yes = 1  Incomplete = 0.5 No/unclear = 0 | **Valid**  Remote sensing = 1 Met station = 0.5 | **Reliable**  Yes = 1  No = 0 | 3 |
| 7. Were important confounding variables measured and adjusted statistically for their impact on the relationship between exposure(s) and outcome(s)? | Yes (env^**^+sociodemographic+IRS+bednets) = 3  Partially (no IRS or no sociodemographic) = 2 No = 0 | | | 3 |
| 8. Was appropriate statistical analysis used including adjusting for temporal/spatial correlation? | Yes = 3  No explicit evaluation of the model = 2  No justification of the model = 1.5  No adjustment for spatial and/or temporal clustering = 1 No = 0 | | | 3 |
| 9. Were results clearly specified (point estimate, confidence intervals, (standard error))? | Excellent = 2  Good = 1.5  Fair = 1  Insufficient = 0.5  No = 0 | | | 2 |
| **Total** |  | | | **22** |

*MIS: Malaria indicator survey

** Environment
