# Supplementary material for: The associations between malaria, interventions, and the environment: a systematic review and meta-analysis
Source: Malar J. 2018 Feb 7;17:73. doi: 10.1186/s12936-018-2220-x (PMC5803989; doi:10.1186/s12936-018-2220-x)
Supplement: Supplementary file 3 — Additional file 3. Characteristics of studies included in the review (n = 11). [file 12936_2018_2220_MOESM3_ESM.docx]

**Additional File 3**

**Characteristics of studies included in the review (n=11)**

| **Authors** | **Country** | **Population and settings** | **Malaria outcome** | **Environmental data** | **Data source** | **Time-frame of observed data** | **Interventions** | **Analysis** | **Results** |
| --- | --- | --- | --- | --- | --- | --- | --- | --- | --- |
| Adigun, A. B., et al. (2015) | Nigeria | Dataset of malariometric information, demographic characteristics and socio-economic status on a nationally representative sample of 5,043 children under five from around 6,000 households from about 240 clusters of which 83 are in the urban areas. | Prevalence (based on the blood slide microscopy) | . Biweekly Normalized Difference Vegetation Index (NDVI) . Decadal rainfall, mm | Remote sensing | October 2009 to October 2010 | Proportion with access to ITN in the household | Bayesian geostatistical logistic regression models | Variation in intervention coverage appears not to be associated with parasitaemia risk. NDVI and rainfall are the most important drivers of malaria risk. |
| Bennett, A. F. Et al. (2016) | Zambia | Three national cross-sectional household surveys in Zambia (2006, 2008, 2010), sub-national prevalence surveys , and Health Management Information System (HMIS) confirmed malaria outpatient incidence data from 2009 to 2011 ; The 2006 MIS covered 1,787 children under 5 in 109 clusters; The 2008 MIS covered 3,010 children under 5 in 169 clusters; The 2010 MIS covered 3,423 children under 5 in 178 clusters. | Parasite prevalence (confirmed cases by microscopy and rapid diagnostic testing RDTs) | . Rainfall (20 days rainfall,mm) . Enhanced vegetation index (EVI) | Remote sensing | 2006, 2008, 2010 | ITN ownership, IRS | Geostatistical models | Climate factors explained the majority of interannual variation in parasite prevalence, but declines in ITN coverage in some high transmission areas led to greater resurgence in 2010 than if coverage levels had been maintained. |
| Chirombo, J., et al. (2014) | Malawi | 3,500 households selected for data collection. Two stage cluster sampling : The first stage selected 140 enumeration areas (EAs) of which 96 were from rural areas and 44 from urban centres. At the second stage, 25 households per EA were selected. | Prevalence (based on rapid diagnostic testing) | . Rainfall (Mean rainfall (mm/day)) . 3 months minimum temperature (°C) | Weather stations | 2010 | ITN use | A structured additive logistic regression model | Minimum temperature and rainfall were not significantly associated with malaria. Children sleeping under an ITN had 43% lower odds of contracting malaria (adjusted OR=0.57, CI: 0.43, 0.76) compared to those not sleeping under ITN. |

**Characteristics of studies included in the review (n=11)**

| **Authors** | **Country** | **Population and settings** | **Malaria outcome** | **Environmental data** | **Data source** | **Time-frame of observed data** | **Type of interventions** | **Analysis** | **Results** |
| --- | --- | --- | --- | --- | --- | --- | --- | --- | --- |
| Dhimal, M., et al. (2014) | Nepal | Data on the population at risk of malaria at both village development committee (VDC) and district levels were obtained from the Epidemiology and DiseaseControl Division | Annual incidence of P.falciparum (Confirmed cases by microscopy and RDT kits) | . Monthly minimum temperature (°C) . Relative humidity | Weather stations | 2004 to 2012 | IRS, LLIN coverage | Generalized additive mixed models (GAMM) | Only minimum temperature and average RH were significant predictors of malaria incidence. 1°C increase in minimum temperature increased malaria incidence by 27% (RR = 1.27, 95% CI = 1.12-1.45) and a 1% increase in mean RH decreased malaria incidence by 9% (RR = 0.91, 95% CI = 0.83-1.00). Malaria incidence was reduced by 25% per one unit increase of LLINs (RR = 0.75, 95% CI = 0.62-0.92).The effect of both rounds of IRS was not significantly associated with malaria incidence. |
| Diboulo, E., et al. (2016) | Burkina Fasso | A random sample of 574 (176 and 398 respectively in urban and rural settings) clusters and 15,000 households were selected | Parasitaemia (microscopy confirmed) | . 8 days night land surface temperature | Remote sensing | April 2010 to January 2011 | ITN use, IRS and ACT | Bayesian geostatistical logistic regression models | LSTN (Night Land Surface Temperature) is one of the most important factors related to parasitaemia risk. None of the health intervention measures is an important predictor of parasitaemia risk at national level. ACT coverage appears to be an important health system component associated with decreased malaria parasitaemia risk in a number of the health districts |

**Characteristics of studies included in the review (n=11)**

| **Authors** | **Country** | **Population and settings** | **Malaria outcome** | **Environmental data** | **Data source** | **Time-frame of observed data** | **Type of interventions** | **Analysis** | **Results** |
| --- | --- | --- | --- | --- | --- | --- | --- | --- | --- |
| Giardina, F., et al. (2012) | Senegal | 4138 children between 6 and 59 months from 320 clusters and 9600 households was selected through a stratified two-stage sampling procedure. The sampling procedure was stratified by the area type (urban/rural) of the clusters: 67.5% of the selected ones were in rural areas and 32.5% in urban areas. At the second sampling stage 30 households were selected randomly from each cluster. | Confirmed cases (microscopy examination) | . Weekly night land surface temperature (LST)  . Biweekly normalized difference vegetation index (NDVI) | Remote sensing | 2008 to 2009 | Presence of at least one bed net per 2 HH mbrs | Geostatistical Zero-Inflated Binomial models (ZIB) | The posterior estimate of the OR indicates a positive association between NDVI, night LST and parasitaemia, however the corresponding 95% credible intervals include one. Living in a household with at least one ITN per every two members was found to have a protective effect on parasitaemia, reducing the odds by 86% (95% BCI: 30%–97%). |
| Gosoniu, L., et al. (2012) | Tanzania | A two-stage sampling approach was used to select the surveyed population. In the first stage were selected 475 clusters consisting of enumeration areas defined for the 2002 Population and Housing Census" The second stage of selection involved the systematic sampling of households from the clusters. A total of 9144 households throughout Tanzania were sampled - 6360 children under 5 | Parasitaemia prevalence (Blood samples testing) | . Annual average day land surface temperature (<26°C, 26-29°C, 29°C) . Annual average night land surface temperature (<16°C, 16-20°C, 20°C) . Annual average rainfall (< 15mm, 15-20 mm, 20mm) . Annual average normalized difference vegetation index (< 0.4, 0.4- 0.6, 0.6) | Remote sensing | October 2007 to February 2008 | IRS activities and ownership of bednets or ITNs | Bayesian geostatistical models | When all covariates were included in the model, only residence, age, wealth index (less poor, least poor) remained statistically significant associated with parasitaemia risk in mainland Tanzania.  Intervention measures (bednet, IRS) seem to have no statistically significant effect on malaria risk. |
| Graves, P. M., et al. (2008) | Eritrea | Clinically diagnosed data of under and over 5 years, from National Health Information system and National Malaria Control Programme | Monthly clinical diagnosis cases (no microscopy and rapid tests) | . Rainfall (monthly precipitation, mm/day) . Maximum value of 30 days normalized Difference Vegetation Index (NDVI) | Satellite | 1998 to 2003 | Monthly numbers of new and old nets impregnated, IRS, larval control | Poisson regression model | Malaria cases were significantly associated with climate variables, although more strongly with the vegetation index NDVI in current and last month than with actual rainfall. When controlling for the effects of interventions, the influence of rainfall and NDVI was changed in the less endemic area. |

**Characteristics of studies included in the review (n=11)**

| **Authors** | **Country** | **Population and settings** | **Malaria outcome** | **Environmental data** | **Data source** | **Time-frame of observed data** | **Type of interventions** | **Analysis** | **Results** |
| --- | --- | --- | --- | --- | --- | --- | --- | --- | --- |
| Lowe, R., et al. (2013) | Malawi | Population under five years and five years and over, for the period July 2004 - June 2011. The population figures for the districts were obtained from the population projections report by the NSO based on the 1998 population and housing census. | aggregated monthly clinically and non-clinically diagnosed malaria cases | . Monthly precipitation estimates (mm/day) . Temperature estimates (°C) | Satellite | July 2004 to June 2011 | ITN distribution rate | Generalized linear mixed model | Once confounding factors were accounted for, a quadratic relationship between temperature and malaria risk in Malawi was not found to be statistically significant. As in the fixed effects model, the number of health facilities per inhabitant was positively associated to malaria relative risk, as was the ITN distribution rate. |
| Riedel, N., et al. (2010) | Zambia | 120 standard enumeration areas were randomly selected among about 17,000 SEAs the country is divided. Within each SEA, a random sample of 25 households was chosen resulting in a total of 3,000 households, 1324 children under five years of age, at 109 cluster locations had complete parasitological data linked to a geo-located household | Malaria parasitemia (blood examination) | . 8 days land surface temperature day and night . 16 days average normalized difference vegetation index  . Daily rainfall estimates | Remote sensing | June 2006 | Presence of at least one bed net in HH, IRS | Multivariate logistic regression models | When assessing the effects of interventions, lack of significance of the environmental factors appears, partially explained by a stronger influence of interventions' effects on the parasitaemia risk than the environmental factors. Interventions are a major driver of parasitaemia risk in Zambia. |
| Thomson, M. C., et al. (1999) | Gambia | 2,276 children (1–4 years of age) from 65 villages from 5 ecologically different areas | Malaria prevalence (blood sample examination) | . 10 days Normalized Difference Vegetation Index (NDVI) | Satellite | 1992 to 1995 | Bednets use | Logistic regression model | The associations with bed net use (treated or untreated) and with the satellite data (NDVI) are significant at the 1% and 5% levels, respectively. |
